# Reductive activation of the disulfide-containing antibiotic thiolutin is mediated by both bacillithiol and FAD-dependent disulfide reductases

**DOI:** 10.1128/jb.00181-25

**Published:** 2025-07-03

**Authors:** Ahmed Gaballa, Yesha Patel, John D. Helmann

**Affiliations:** 1 Department of Microbiology, Cornell University 251789, Ithaca, New York, USA; University of Notre Dame, Notre Dame, Indiana, USA

**Keywords:** dithiolopyrrolone, antibiotic, thiolutin, holomycin, zinc, thioredoxin reductase, bacillithiol

## Abstract

Metal ions are universally essential for life and are required for critical enzymes throughout metabolism. Metalloenzymes rely on metal import and trafficking pathways for loading of the desired metal. Many microbes produce natural products that serve as metal chelators, both for their own nutrition and to serve as antimicrobials. In response to infection, our immune cells restrict bacterial growth by deploying proteins that chelate metal ions as part of nutritional immunity. Cells respond to metal depletion by the activation of pathways that prioritize metal delivery to the most essential enzymes. Dithiolopyrrolone (DTP) class natural products are prodrugs that are reduced in cells to generate a potent, dithiol-containing zinc chelator. Here, we identify the cellular reductants, bacillithiol and the FAD-dependent oxidoreductases TrxB and AhpF, that activate the DTP antibiotic thiolutin in *Bacillus subtilis*. Genetic studies reveal that loss of the Spx transcription factor also increases thiolutin resistance, consistent with the known role of Spx in transcriptional activation of thioredoxin reductase (*trxB*) and genes required for bacillithiol synthesis. Collectively, our results support a model in which several parallel pathways all contribute to the reductive activation of DTP class prodrugs *in vivo*.

**IMPORTANCE:**

Metal ion chelators (metallophores) are deployed by microbes to obtain nutrient metals, sequester excess metals, and act as antimicrobials to inhibit the growth of other organisms. Dithiolopyrrolones (DTPs) are a class of natural products that inhibit bacterial growth by the intracellular chelation of zinc and iron, two metal ions essential for growth. Thiolutin, a model DTP antibiotic, is activated by reduction inside cells and selectively chelates intracellular metals. Here, we demonstrate that the activation of the thiolutin prodrug is mediated by several parallel pathways, which greatly reduces the ability of cells to evolve antibiotic resistance. Since DTP antibiotics appear to primarily target zinc enzymes, they provide a powerful tool for exploring how cells adapt to zinc deficiency.

## INTRODUCTION

The gram-positive bacterium, *Bacillus subtilis*, is a model system for defining mechanisms of bacterial metal homeostasis. Metalloregulators play a key role in this process by differentially regulating genes to improve growth under conditions of metal deficiency or excess. The genetic responses to changes in metal availability often occur in a graded (or stepwise) fashion (1). For example, zinc (Zn) homeostasis is coordinated in a graded manner by the Zur metalloregulator (2). As cells transition from Zn sufficiency to deficiency, there is sequential derepression of (i) Zn-independent L31 and L33 ribosomal proteins (3, 4), (ii) the high-affinity Zn uptake system ZnuABC and Zn-metallochaperone ZagA (5, 6), and finally, (iii) the Zn-independent S14 ribosomal protein (7) and GTP cyclohydrolase IB, FolEB (8). Adaptations to changing iron (Fe) and manganese (Mn) levels also follow a graded response (9–11).

Studies to identify the genes induced by changes in metal availability, and the order of their induction, require a means to control intracellular metal levels. The simplest approach is to vary the metal present in the growth medium, with low levels or omission of a nutrient metal eliciting an adaptive response. In *B. subtilis*, this approach works well for monitoring Mn requirements (10), but not for studies of Zn physiology (3). Studies of bacterial Zn physiology are challenging because high-affinity import systems (ZnuABC) allow growth even in the absence of added Zn, presumably because contaminating Zn is sufficient to support growth (12). With the use of uptake-deficient mutant strains, Zn-restricted growth can be reproducibly achieved by careful control of the Zn content of the growth medium (3).

An alternative approach to induce metal deficiency relies on metal chelators. For studies of Zn physiology, EDTA can be used to deplete extracellular Zn, and N,N,N′,N′-tetrakis(2-pyridinylmethyl)-1,2-ethanediamine (TPEN) serves as a cell-permeable Zn chelator. However, chelators often exhibit high affinity for multiple divalent cations, leading to off-target effects. Although TPEN is useful for studies of Zn physiology, it can deplete both extracellular and intracellular Zn and affect Fe pools (2, 13).

**Fig 1:**
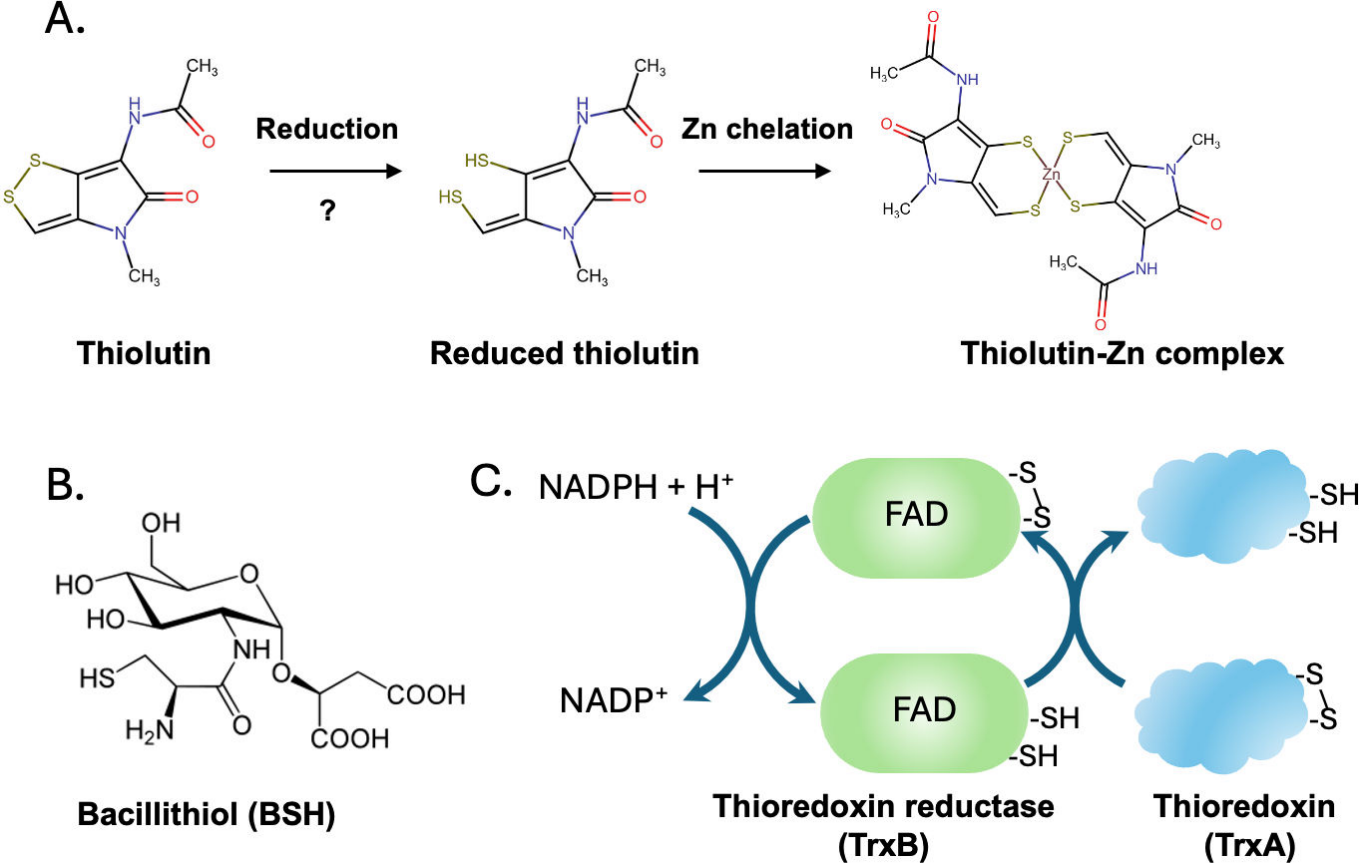
The intracellular reduction of DTP antibiotics. (**A**) The DTP antibiotic thiolutin is reduced to its active dithiol form by cellular reductants. Reduction of DTP antibiotics to their active form may involve (**B**) the abundant low molecular-weight thiol bacillithiol (BSH) and (**C**) the abundant, redox-active small protein thioredoxin (TrxA) and/or the cognate thioredoxin reductase (TrxB). TrxB is an NADPH-dependent, FAD-containing disulfide reductase that maintains the TrxA pool in a reduced state.

Dithiolopyrrolones (DTPs) are antibiotics that provide a strategy to specifically deplete intracellular metals (14–16). DTPs are microbially produced secondary metabolites that exhibit broad-spectrum antibiotic activity against gram-positive and gram-negative bacteria, as well as potent anti-cancer activity (14). The first described DTP antibiotics were thiolutin and aureothricin, natural products isolated from *Streptomyces* spp., beginning in 1948. Structural comparisons revealed that both compounds had a common dithiolopyrrolone structure that provides the name for this class of antibiotics (17). Holomycin differs from thiolutin only by the replacement of the pyrrolone methyl group with hydrogen and has also served as a model compound for this class of antibiotics (16, 18–20). More than a dozen related compounds have since been described from both gram-positive and gram-negative bacteria (14). DTP antibiotics are prodrugs activated in cells by reduction to their active dithiol form (Fig. 1A). Studies in *Escherichia coli* have shown that the reduced form of the DTP antibiotic holomycin is a metal chelator that inhibits Zn enzymes and some enzymes with FeS clusters (16, 19).

The pathways that mediate the activation of DTP antibiotics in cells have been controversial. Reduction of holomycin may be mediated by glutathione (21), the major low molecular-weight thiol in many cells. However, reduction is slow and requires a large molar excess of glutathione (20, 21). In many bacteria, glutathione is functionally replaced by bacillithiol (BSH), a conjugate of D-glucosamine with cysteine and L-malic acid (Fig. 1B) (22–24). At high concentrations, BSH also reduces holomycin *in vitro*, and the selection of *Staphylococcus aureus* strains with high-level resistance to the bifunctional DTP antibiotic thiomarinol led to strains with a mutation in *bshA*, a gene for BSH synthesis (20). However, these evolved strains each had a minimum of seven different mutations, and their overall effect on holomycin resistance was comparatively modest (fourfold). Resistance to thiolutin has also been investigated in *Saccharomyces cerevisiae*, where it was found that changes in thioredoxin reductase and thioredoxins affected sensitivity (25). These studies led to the suggestion that thioredoxins, small proteins that contain a redox-active Cys-x-x-Cys dithiol motif that maintains protein thiols in a reduced state (Fig. 1C), might also reduce thiolutin. Alternatively, thioredoxin reductase (TrxB) and homologous NADPH-dependent reductases may directly reduce DTP antibiotics, as supported by biochemical studies of holomycin reduction (20). *B. subtilis* contains TrxB and two other related disulfide reductases: AhpF partners with AhpC to reduce peroxides (26, 27) and Bdr reduces bacillithiol disulfide (BSSB) (28–31). However, no single enzyme was required for holomycin activity *in vivo*, suggesting that there are multiple parallel pathways for antibiotic reduction (20).

Here, we investigated the pathways involved in the intracellular activation of the DTP antibiotic thiolutin. Using a combination of forward and reverse genetics, we demonstrate that in *B. subtilis*, the low molecular-weight thiol BSH, TrxB, and alkyl hydroperoxide reductase (AhpF) all contribute to activation *in vivo*. We additionally found that mutations affecting Spx, a global redox-activated regulator that activates both BSH synthesis and the thioredoxin system (32), can also confer modest resistance to thiolutin. Consistent with studies on the reduction of DTP antibiotics in *S. aureus* (20), we conclude that activation of thiolutin occurs by multiple independent pathways, with BSH, TrxB, and AhpF all contributing to prodrug reduction.

## RESULTS

### BSH contributes to thiolutin sensitivity *in vivo*

**Fig 2:**
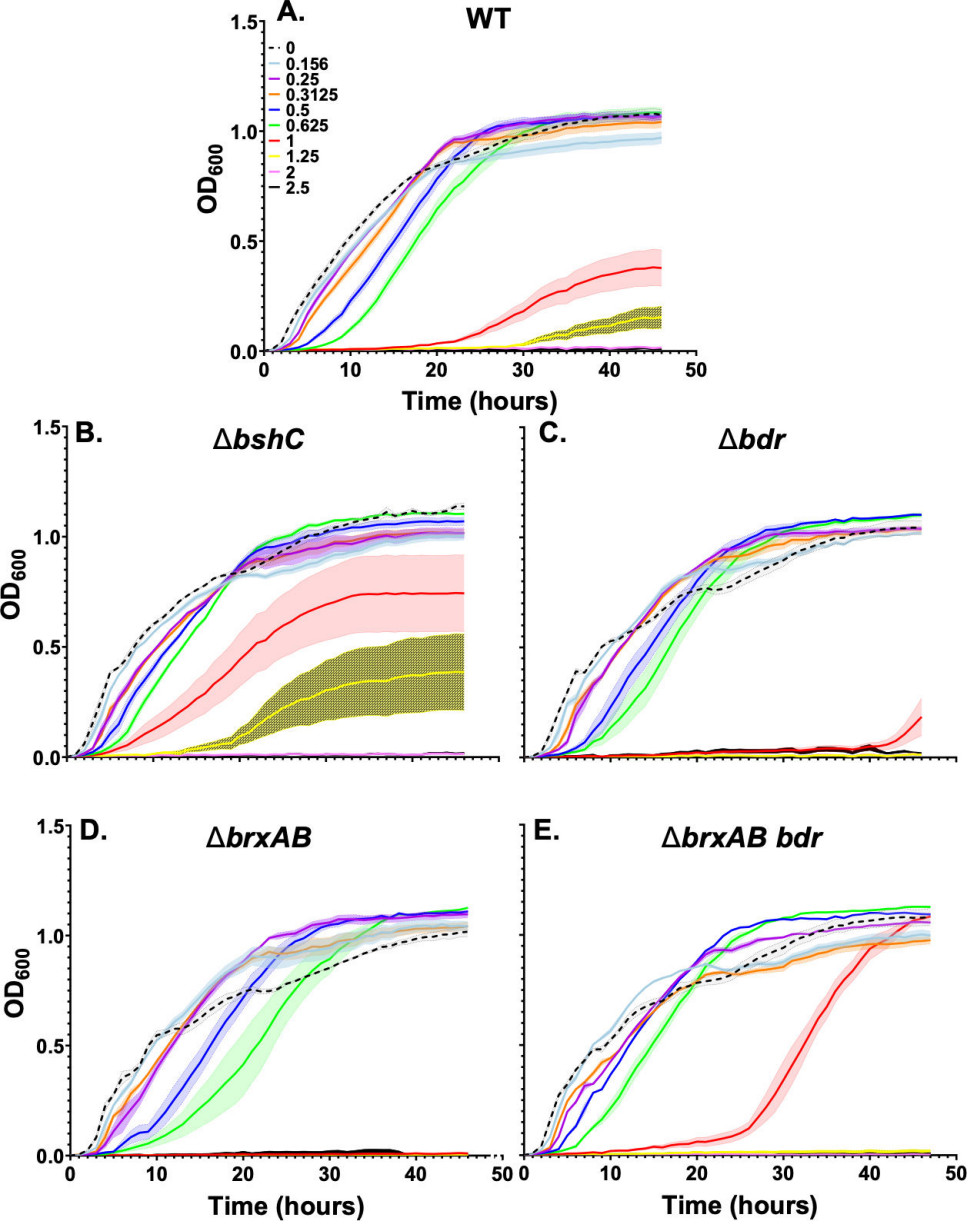
Growth of *B. subtilis* strains: (**A**) wild type (WT), (**B**) Δ*bshC*, (**C**) Δ*bdr*, (**D**) Δ*brxA* Δ*brxB* (Δ*brxAB*), and (**E**) Δ*brxA* Δ*brxB* Δ*bdr* (Δ*brxAB bdr*) in the presence of thiolutin (0–2.5 µg/mL). Results are the average of at least three biological replicates, and the shaded region denotes the standard error of the mean. Note that for these and subsequent growth curves, up to 200 cultures were monitored in parallel in a single experiment, and some curves are shown in multiple panels for ease in comparison.

To understand the role of intracellular BSH in thiolutin activation, we compared the effects of thiolutin on the growth of cells lacking BSH (∆*bshC*) with their isogenic parent strain (wild type [WT]) (Fig. 2A and B; Fig. S1). The monocistronic *bshC* gene encodes the last enzyme in BSH biosynthesis. The ∆*bshC* strain lacks BSH, and this defect can be complemented by the expression of *bshC* from an ectopic locus (33). The ∆*bshC* mutant was significantly more resistant to thiolutin, an effect most noticeable in the growth curves in the presence of 1 µg/mL thiolutin. At this concentration, ∆*bshC* had a much shorter growth lag and an increase in yield (Fig. 2A and B). This suggests that BSH contributes, either directly or indirectly, to the activation of thiolutin *in vivo*.

**Fig 3:**
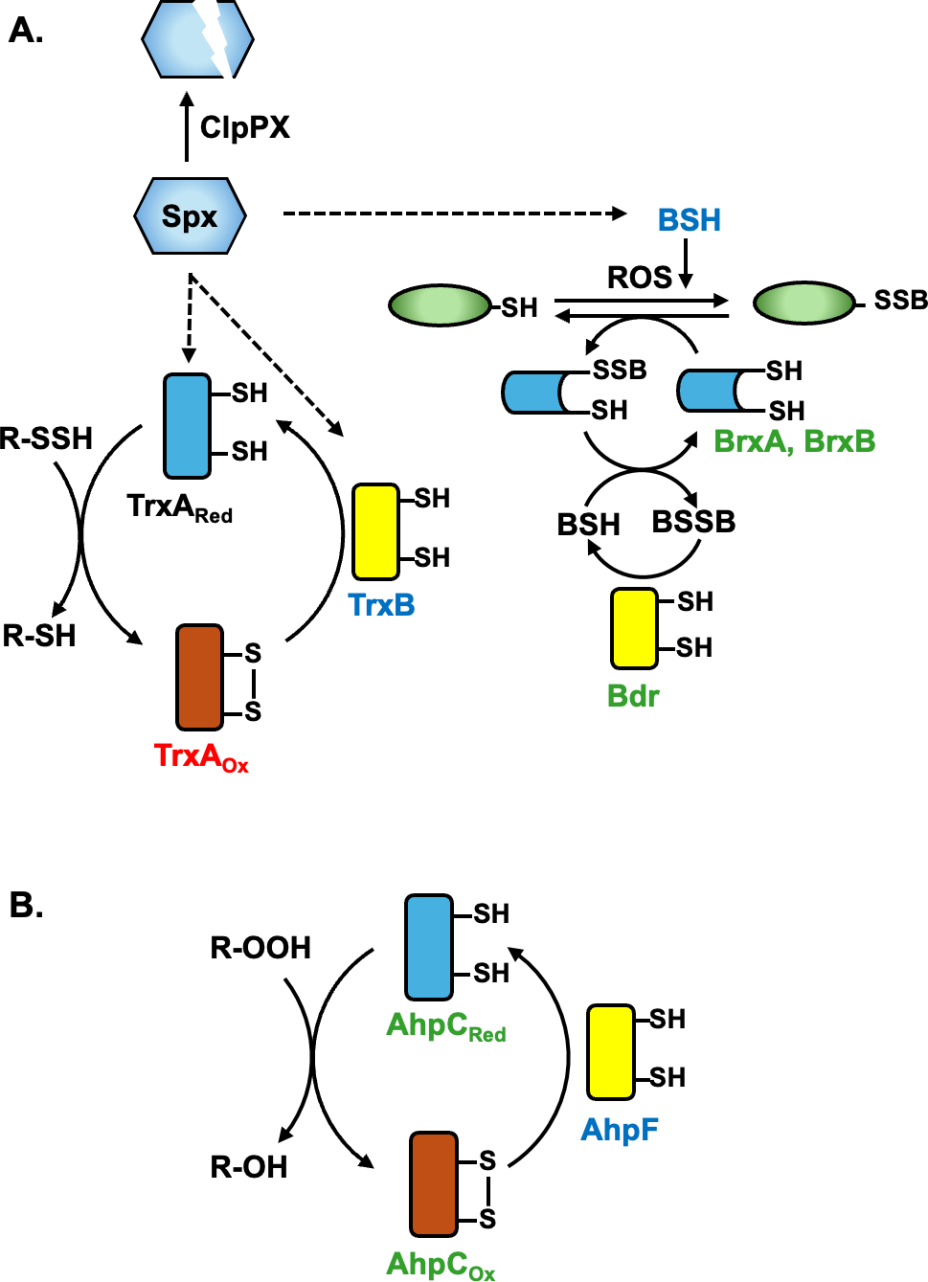
Schematic diagram of *B. subtilis* reductants. (**A**) The redox-sensitive transcription factor Spx activates transcription of *trxA* and *trxB* (encoding thioredoxin-thioredoxin reductase; TrxAB) and the genes for BSH synthesis. TrxB is an NADPH-dependent FAD-containing disulfide reductase (yellow fill). *B. subtilis* uses bacillithiol (BSH) as the major low molecular-weight thiol to maintain intracellular proteins in their reduced state. S-bacillithiolated proteins (green fill) can be de-bacillithiolated by C-x-C-containing bacilliredoxins (BrxA and BrxB; blue fill) (28), a process that leads to the formation of oxidized bacillithiol (BSSB). BSSB is reduced using the TrxB paralog, bacillithiol disulfide reductase (Bdr; yellow fill) (28, 29). In addition, *B. subtilis* expresses (**B**) alkyl hydroperoxide reductase, which consists of a small redox active protein (AhpC) and the TrxB paralog, AhpF (yellow fill). Font colors summarize the inferred role in thiolutin reduction *in vivo* based on the results reported herein: BSH, TrxB, and AhpF are implicated as reductants (blue); AhpC, Bdr, BrxA, and BrxB had little effect on thiolutin activity (green); TrxA, likely in its oxidized form (TrxA_ox_), confers resistance (red).

We next tested the possible roles of other BSH-related genes (Fig. 3) in thiolutin reduction. We hypothesized that thiolutin reduction might be mediated by BSSB oxidoreductase (Bdr; previously known as YpdA), a member of the NADPH-dependent, FAD-containing disulfide reductase family (28, 29, 31). If Bdr activates thiolutin, we predicted that a ∆*bdr* strain might be more resistant. However, the ∆*bdr* strains displayed an increased sensitivity relative to WT at 1 µg/mL of thiolutin (Fig. 2C). *B. subtilis* encodes two bacilliredoxins, BrxA and BrxB (UNIPROT uncharacterized protein family; UPF0403), with a Cys-Gly-Cys redox motif that reduces disulfides in S-bacillithiolated cytosolic proteins (28, 30). The ∆*brxA* ∆*brxB* double mutant was also more sensitive to thiolutin than WT (Fig. 2D). For reasons not yet clear, the ∆*brxA* ∆*brxB* ∆*bdr* triple mutant (Fig. 2E) showed enhanced growth at 1 µg/mL thiolutin compared to ∆*brxA* ∆*brxB* and ∆*bdr* mutants and a higher yield compared to WT (Fig. 1). We conclude that neither the bacilliredoxins (BrxA and BrxB) nor the Bdr oxidoreductase plays a significant role in the activation of thiolutin. BSH does contribute to thiolutin activation *in vivo*, an effect likely mediated by direct reduction, as seen *in vitro* (20). However, thiolutin still inhibits the growth of the ∆*bshC* strain, which implies that there are other pathways for thiolutin activation.

### Mutations in *spx*, *ahpC*, and *trxA* affect thiolutin resistance

To identify additional pathways for thiolutin reduction, we used a forward genetics approach to isolate mutant strains with reduced susceptibility to thiolutin. WT and ∆*brxA* ∆*brxB*, ∆*bdr*, and ∆*brxA* ∆*brxB* ∆*bdr* mutants were grown in replicate in microtiter plates in lysogeny broth (LB) medium amended with different concentrations of thiolutin. After prolonged incubation, we noticed sporadic growth in wells with an inhibitory concentration of thiolution (2.5 µg/mL). After colony purification, we confirmed the resistance phenotype and found that, similar to the ∆*bshC* mutant, the isolated strains had a modest but reproducible increase in thiolutin resistance (Fig. S2).

Next, we used whole-genome resequencing to identify the genetic changes in six isolates with increased thiolutin resistance (Table 1). Each mutant strain had acquired one or two single nucleotide variants (SNVs). In three different strain backgrounds, we recovered nonsense mutations in *spx*. Spx is a redox-responsive transcription regulator widely disseminated in low GC gram-positive Bacillota (32). The other changes included a missense mutation affecting alkyl hydroperoxide reductase (AhpF) and one affecting thioredoxin (TrxA). A role for TrxA was further supported by the recovery of a C>T mutation in the promoter region of *trxA*. Mutations were also noted in two strains in a non-coding region near the S169 RNA element that is antisense to the integrase (*int*) gene of an integrative-conjugative element (ICE*Bs1*; 34) and a silent mutation in codon 2 of the uridine kinase (*udk*) gene (Table 1). We hypothesized that the mutations in *spx*, *ahpF*, and *trxA* most likely account for thiolutin resistance as these genes are all implicated in pathways related to thiol oxidation and reduction (Fig. 3).

**TABLE 1:** List of mutations in suppressor strains with reduced thiolutin sensitivity

Suppressor	Background strain	Genomic change (locus, SNV related to WT)	Gene	Amino acid
1	WT	1227753, G>A	*spx*	Trp19*^b^
2	∆*brxA* ∆*brxB*	529446, C>T	3′ of *trnS*-Leu2	NA
		4121074, G>T	*ahpF*	Gly354Val
3	∆*bdr*	1227889, C>T	*spx*	Gln65*^b^
		2792848, A>C	*udk*	Silent (Gly2)
4	∆*brxA* ∆*brxB* ∆*bdr*	529455, A insertion	S169 (3′ of *trnS*-Leu2)	NA
		2913184, A>T	*trxA*	Ile52Asn
5	∆*brxA* ∆*brxB* ∆*bdr*	1227757, G>T	*spx*	Glu21*^b^
6	∆*brxA* ∆*brxB* ∆*bdr*	2913380, C>T	P*_trxA_*^*a*^	NA

^*a*^ This substitution changes the extended −10 region from **TG**C**TATA**C**T** (bold residues match consensus) to **T**AC**TATA**C**T**.

^*b*^ Asterisks indicate a stop codon.

### The Spx transcription factor contributes to thiolutin sensitivity

Spx is a global regulator that controls the expression of a large regulon of at least 52 genes (35) and many more once activated by stresses such as oxidants (e.g., diamide) or cell wall antibiotics (36, 37). Expression of *spx* is under complex transcriptional control (38–40) and is regulated by oxidation of a redox-active Cys_10_-x-x-Cys_13_ motif and by proteolysis (41, 42).

**Fig 4:**
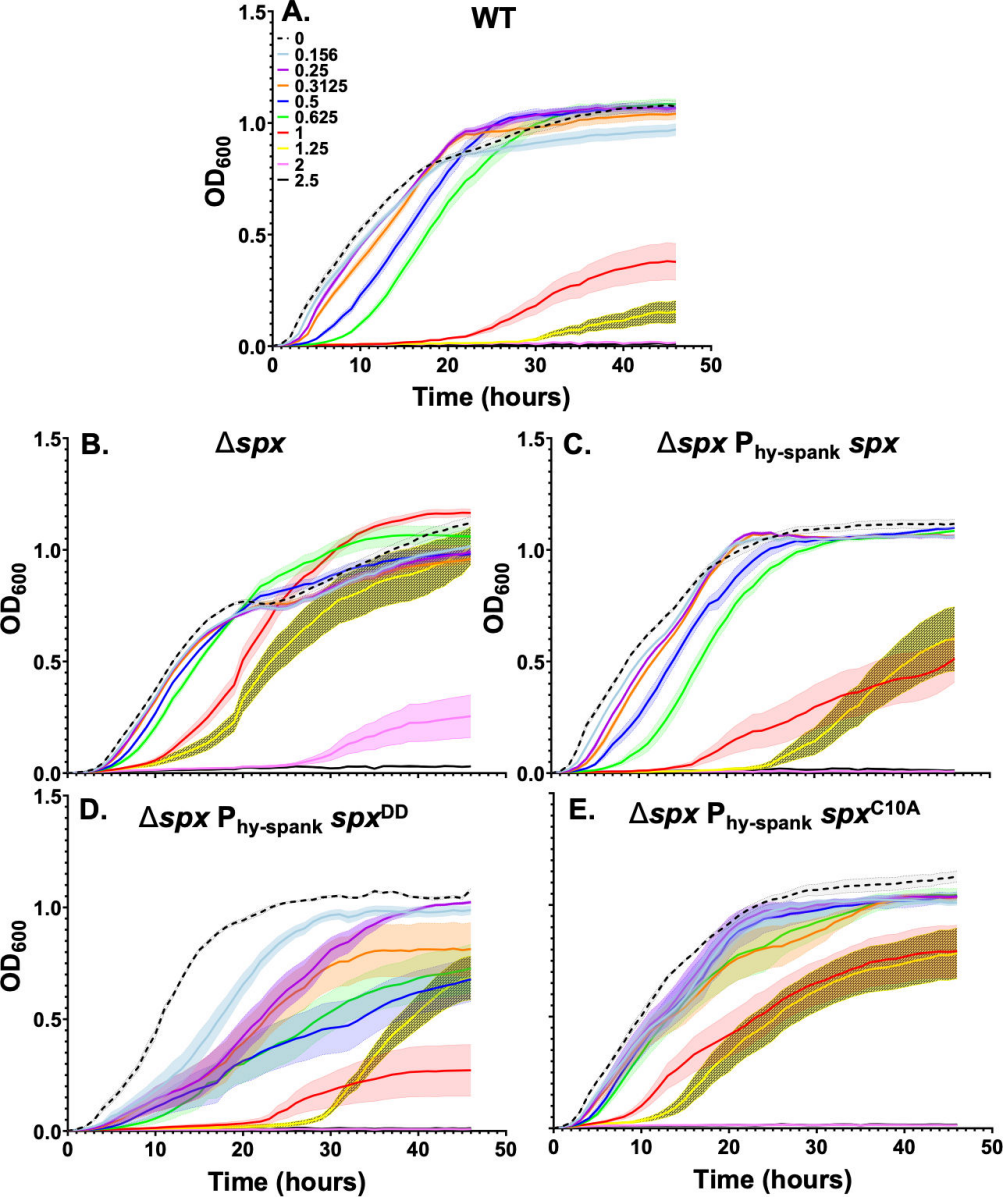
Growth of *B. subtilis* strains: (**A**) WT, (**B**) Δ*spx*, (C–E) Δ*spx* complemented with an IPTG-induced ectopic copy encoding (**C**) Spx, or (**D**) Spx DD, or (**E**) Spx C10A in the presence of thiolutin (0–2.5 µg/mL). Results are the average of at least three biological replicates, and the shaded region denotes the standard error of the mean.

We compared the thiolutin sensitivity of WT cells with a ∆*spx* mutant and a complemented strain. As expected, the ∆*spx* mutant (Fig. 4B) was more resistant to thiolutin than WT (Fig. 4A), and this phenotype was complemented by the expression of an Isopropyl β-D-1-thiogalactopyranoside (IPTG)-inducible *spx* allele (Fig. 4C). We conclude that Spx contributes to thiolutin sensitivity, likely through its effects on transcription. A protease-resistant Spx^DD^ mutant protects Spx against ClpXP-mediated proteolysis (Fig. 3), thereby upregulating Spx-dependent genes (43). As expected, IPTG-induced expression of Spx^DD^ strongly sensitized the ∆*spx* mutant to thiolutin (Fig. 4D).

### A reduction in BSH synthesis is insufficient to explain the effect of Spx on thiolutin sensitivity

Spx activates transcription of the genes for BSH synthesis (*bshA*, *bshB1*, *bshB2*, *bshC*) (44). In a ∆*spx* strain, BSH levels are reduced ~3–4-fold with ~25% residual BSH remaining (44). If ∆*spx* confers thiolutin resistance solely through a reduction in BSH levels, we reasoned that a ∆*bshC* mutant should be more resistant than a ∆*spx* mutant. However, the ∆*bshC* mutant was significantly less resistant than the ∆*spx* strain (Fig. 4B vs Fig. 2B; Fig. S1). We conclude that the reduced levels of BSH in the ∆*spx* background can only partially explain thiolutin sensitivity (Fig. 3A).

Although the Spx regulon is large (36), *trxA* and *trxB* are among the most strongly regulated targets of Spx activation and are very poorly transcribed in a ∆*spx* strain (43, 45). Previously, it was found that Spx C10A, lacking a redox-active cysteine residue, prevents the diamide induction of *trxA* and *trxB* (46), but not *bshC* (44). Expression of an IPTG-induced Spx C10A mutant protein failed to restore thiolutin resistance to a ∆*spx* strain (Fig. 4E). This suggests that redox activation of Spx is important for conferring thiolutin sensitivity by activating expression of *trxA* and *trxB* (Fig. 3A).

### Thioredoxin reductase is involved in thiolutin reduction *in vivo*

**Fig 5:**
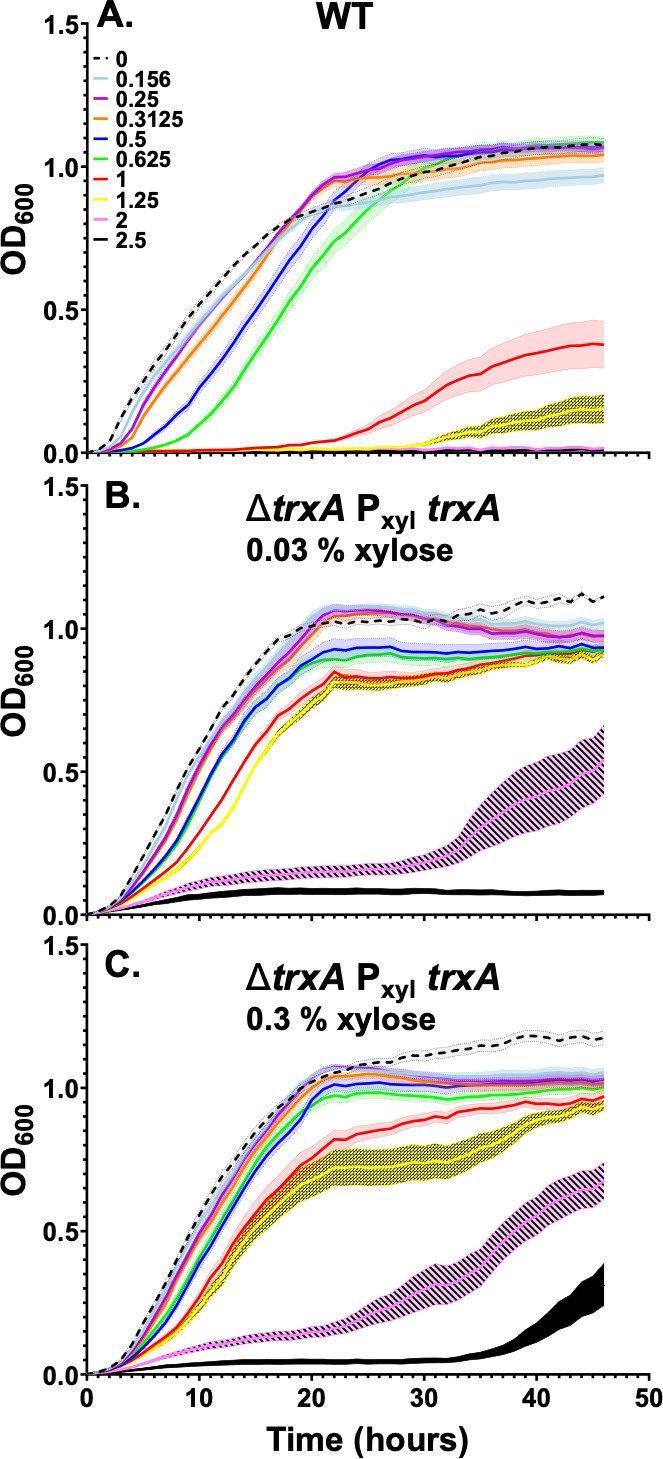
Growth of *B. subtilis* strains: (**A**) WT, (**B**) Δ*trxA amyE*::P_xyl_-*trxA* with 0.03% xylose, and (**C**) Δ*trxA amyE*::P_xyl_-*trxA* with 0.3% xylose. The concentrations of thiolutin ranged from 0 to 2.5 µg/mL. Results are the average of at least three biological replicates, and the shaded region denotes the standard error of the mean.

We next set out to test the role of TrxA and TrxB as possible cellular reductants for thiolutin. Both *trxA* and *trxB* are essential for *B. subtilis*, so we used *trxA* and *trxB* mutants expressing the corresponding gene ectopically under the control of a xylose-inducible promoter (47). Induction of *trxA* increased thiolutin resistance (Fig. 5), suggesting that the mutations we recovered (Table 1) are likely to increase TrxA activity or levels in the cell. In contrast, inducible expression of *trxB* strongly sensitized the cell to thiolutin, although these strains were compromised in growth under our conditions (Fig. S3). These data are consistent with the hypothesis that TrxB can reduce thiolutin *in vivo*. Elevated levels of TrxA might inhibit this process by serving as a competitive inhibitor of TrxB-mediated thiolutin reduction. Alternatively, oxidized TrxA might catalyze the oxidation of thiolutin back to its inactive, dithiol form.

### AhpF plays a minor role in thiolutin reduction *in vivo*

**Fig 6:**
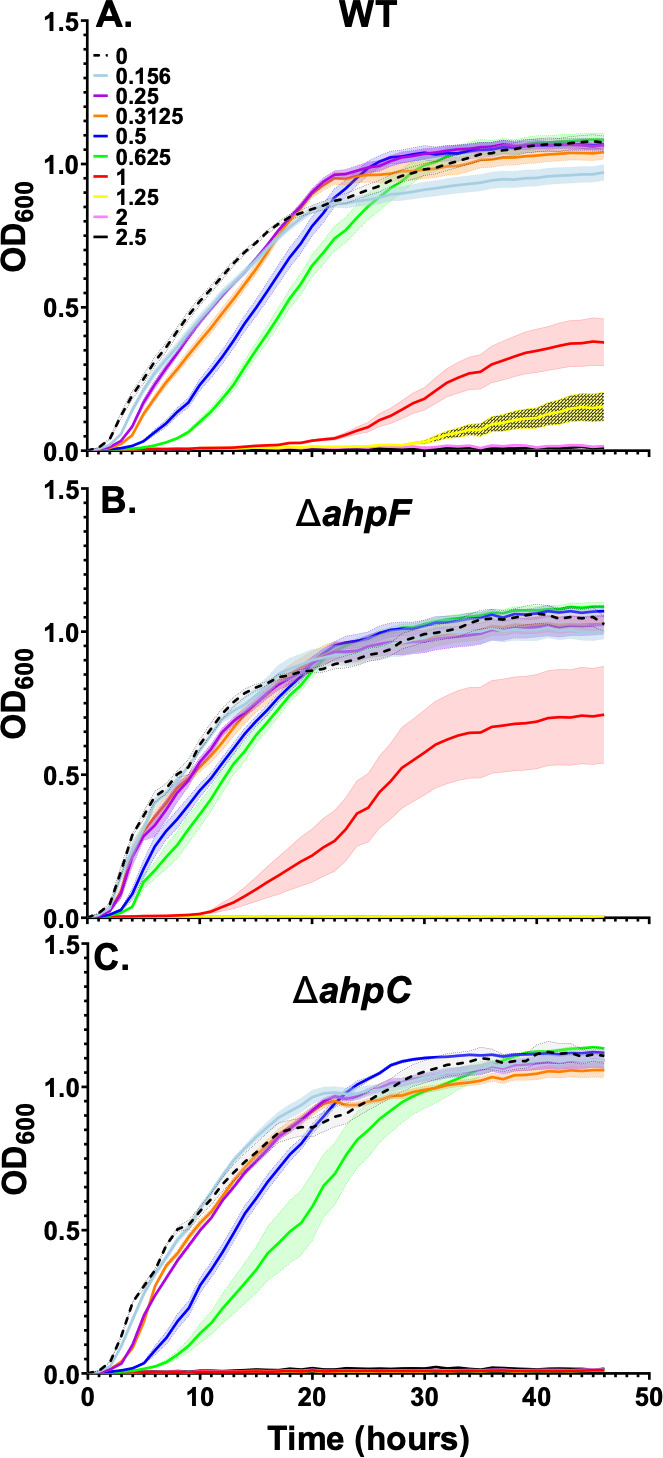
Growth of *B. subtilis* strains: (**A**) WT, (**B**) Δ*ahpF*, and (**C**) Δ*ahpC* in the presence of different concentrations of thiolutin (0–2.5 µg/mL). Results are the average of at least three biological replicates, and the shaded region denotes the standard error of the mean.

In our selection for thiolutin resistance, we isolated a missense G354V mutation in *ahpF* (Table 1; Fig. S3), encoding the large subunit of the alkyl hydroperoxide reductase AhpCF (Fig. 3B). When we analyzed the growth of ∆*ahpC* and ∆*ahpF* mutants in the presence of thiolutin, we found that the ∆*ahpF* mutant reduced the lag phase at 1 µg/mL thiolutin from 20 hours (in WT; Fig. 6A) to 10 hours (Fig. 6B). Hence, it is likely that the G354V mutant (Table 1) reduced the activity of AhpF, and that AhpF can also contribute to thiolutin activation *in vivo*.

We also tested a strain with a disruption of *ahpC* and found that this strain was more sensitive to thiolutin than WT (Fig. 6C). This likely results from increased transcription of the *ahpF* gene. The disruption of *ahpC* inactivates the AhpCF alkyl hydroperoxide reductase, and this leads to derepression of the PerR regulon, which includes the promoter for the *ahpCF* operon (26, 27, 48). In addition, the *ahpC*::*tet* cassette may also drive the expression of the downstream *ahpF* gene. Together, these effects are predicted to increase the level of AhpF, which may thereby sensitize cells to thiolutin.

## DISCUSSION

DTP class antibiotics have a broad spectrum of activity that has been linked to the dysregulation of metal homeostasis (19). Biochemical studies have demonstrated that LMW thiols, such as glutathione, coenzyme A, and BSH, can reduce the DTP ene-disulfide to the active, vicinal dithiol (20, 21). However, the pathways that reduce DTP compounds *in vivo* are less well characterized.

Here, we have used forward and reverse genetic approaches to identify genes that affect the sensitivity of *B. subtilis* to thiolutin. Collectively, our results imply a model in which reduction occurs through multiple, largely independent pathways (Fig. 3). A role for BSH is consistent with an increase in thiolutin resistance in cells lacking BSH (∆*bshC*), and with results from *S. aureus* where mutations in *bshA* were found in strains evolved to be resistant to thiomarinol, a hybrid antibiotic that targets both Ile-tRNA synthetase and metal homeostasis (20). We did not detect a contribution from the BSSB reductase (Bdr), although the homologous enzyme glutathione oxidoreductase (Gor) can reduce holomycin *in vitro* (20). Cells lacking Bdr have little change in intracellular BSH, although they do accumulate higher levels of BSSB (30, 31). Thus, it is most likely that BSH rather than Bdr is directly involved in thiolutin activation. Consistently, purified *S. aureus* Bdr protein did not reduce holomycin *in vitro* (20).

The DTP antibiotic holomycin accumulates in *E. coli* to levels in excess of 1 mM (49). If accumulation is similar in *B. subtilis*, full reduction would require >2 mM BSH. However, the measured levels of BSH during the growth of *B. subtilis* in LB medium vary from ~200 µM during exponential growth to ~1 mM in the stationary phase (24), which could partially but not fully reduce mM levels of DTP antibiotics. BSH also serves as a buffer of intracellular Zn (50). If the reduction of thiolutin depletes the BSH pool, this may lead to a release of BSH-bound Zn that could then be sequestered by binding to thiolutin, thereby further depleting the labile Zn pool that is needed for metalation of Zn-dependent enzymes.

Since cells devoid of BSH (∆*bshC*) have only a modest increase in fitness in the presence of thiolutin (Fig. 2), there must also be other pathways for activation. Genetic analyses of mutant isolates identified loss of function mutations in *spx* and missense mutations *in trxA* and *ahpF* genes that contribute to thiolutin resistance (Table 1). Inactivation of Spx (Fig. 3), which activates both BSH synthesis (44) and TrxA/TrxB (32, 45), provides one pathway for the emergence of resistance (Table 1). Genetic analyses with null mutations and overexpression constructs support a model in which TrxB and AhpF, two FAD-containing thiol oxidoreductases, can also activate thiolutin (Fig. 3). Since the induction of TrxA modestly increases thiolutin resistance (Fig. 5), we suggest that the recovered *trxA* mutations may increase TrxA levels or activity. TrxA is an abundant protein in *B. subtilis*, with ~10^4^ copies per cell and a ratio of ~15 TrxA molecules for every TrxB reductase (51). The TrxA thioredoxin might increase thiolutin resistance by serving as a competitive inhibitor of TrxB-dependent thiolutin activation or, in its oxidized form, by directly oxidizing thiolutin back to its inactive, dithiol form. In sum, our results support the idea that activation of DTP antibiotics is mediated by multiple, parallel pathways (20), and as a result, no single mutation leads to high-level resistance. Indeed, by combining mutations that affect parallel pathways, additive effects on resistance can be seen (Fig. S1).

We are interested in exploring thiolutin as a tool for investigating Zn physiology in *B. subtilis*. Unlike TPEN, which coordinates Zn through nitrogen atoms from four pyridine rings, thiolutin binds Zn via sulfur atoms (14, 16). This difference in coordination chemistry, and a less sterically restricted metal ligand (a thiol compared to a pyridine ring), may allow DTP antibiotics to target a distinct labile metal pool (52) and perhaps affect selected metal-dependent enzymes. However, effects on the BSH pools are a potential confounding factor since BSH itself buffers intracellular Zn and also affects Fe physiology (50, 53).

## MATERIALS AND METHODS

### Strains and growth conditions

Strains were streaked on LB medium with the corresponding selection antibiotics (Table 2). Strains were constructed by transformation of genomic DNA to cells growing in a modified competence medium as previously described (54). For selection, antibiotics were added at the following concentrations: erythromycin (1 µg/mL) and lincomycin (25 µg/mL) (for selecting for *mls* [macrolide-lincosamide-streptogramin B] resistance), spectinomycin (100 µg/mL), chloramphenicol (10 µg/mL), kanamycin (15 µg/mL), and neomycin (10 µg/mL).

**TABLE 2:** Strains used in this study

Strain	Designation	Genotype	Source/reference
CU1065	WT	W168 *att*SPβ *trpC*2	Lab Stock
HB9140	P*_xylA_-trxA*	*amyE*::P*_xylA_-trxA*	Lab Stock, unpublished
HB9141	∆*trxA* P*_xylA_-trxA*	*trxA*::*tet amyE*::P*_xylA_-trxA*	Lab Stock, unpublished
HB11094	∆*ahpC*	*ahpC::tet*	Lab Stock, unpublished
HB11094	∆*brxA* ∆*brxB*	*brxA*::*kan brxB*::*tet*	(55)
HB11187	∆*bdr*	*bdr*::*spc*	(28)
HB11190	∆*brxA* ∆*brxB* ∆*bdr*	*brxA*::*kan brxB*::*tet bdr*::*spc*	(55)
HB11210	∆*bshC* ∆*spx*	*bshC*::*kan spx*::*spc*	Lab Stock, unpublished
HB11291	∆*trxB* P*_xylA_-trxB*	*trxB*::*erm amyE*::P*_xylB_-trxB*	Lab Stock, unpublished
HB11301	P*_xylA_-trxB*	*amyE*::P*_xylB_-trxB*	Lab Stock, unpublished
HB11306	∆*bshC*	*bshC*::*kan*	(33)
HB18802	∆*spx*	*spx*::*spec*	(40)
HB18805	∆*spx*	*spx*::*neo amyE*::P_hy-spank_-*spx*	(43, 56)
HB18806	∆*spx* P_hs-_*spx*^C10A^	spx::*neo* amyE::P_hy-spank_-*spx* C10A (spec)	(43, 56)
HB18807	∆*spx* P_hs-_*spx*^DD^	*spx*::*neo amyE*::P_hy-spank_-*spx*^DD^ (spec)	(43, 56)
BKE40100		*ahpF*::*erm*	(57)
HB31401	∆*ahpF*	*ahpF*::*erm*	This work
HB31402	∆*ahpF* ∆*bshC*	*ahpF*::*erm bshC*::*kan*	This work
HB31403	∆*ahpF* ∆*spx*	*ahpF*::*erm spx*::*spc*	This work
HB31404	∆*ahpF* ∆*bshC* ∆*spx*	*ahpF*::*erm bshC*::*kan spx*::*spc*	This work

For growth studies, cells were cultured in minimal medium (MM) contained 40 mM potassium morpholinopropanesulfonic acid (adjusted to pH 7.4 with KOH), 2 mM potassium phosphate buffer (pH 7.0), glucose (2%, wt/vol), (NH_4_)_2_SO_4_ (2 g/L), MgSO_4_·7H_2_O (0.2 g/L), trisodium citrate dihydrate (1 g/L), potassium glutamate (1 g/L), tryptophan (10 mg/L), 50 µM FeSO_4_, and 1 µM MnCl_2_.

### Thiolutin susceptibility assay

Cells were precultured in MM and incubated at 37°C with shaking to an optical density at 600nm (OD_600_) of ~0.4–0.6, and 2 µL was used to inoculate 200 µL of MM supplemented with different concentrations of thiolutin (Sigma, catalog number: T3450), added to a final concentration of 0–2.5 µg/mL in Honeycomb 2 microplates. Plates were incubated in a Bioscreen Pro automated growth curve machine at 37°C with shaking, and bacterial growth was monitored by measuring the OD_600_ at 15 minute intervals for 2 days. Results are the mean and standard error of the mean of at least three biological replicates. Note that some growth results are shown in multiple figure panels for ease in comparison between strains.

### Selection of mutants with increased thiolutin resistance

One microliter of 0.4 OD_600_ cells of each mutant was inoculated in LB in microtiter plates with increasing concentrations of thiolutin for an extended time (66 hours). This prolonged incubation allowed mutations to arise that could help the cells tolerate high concentrations of thiolutin. Under these conditions, growth was observed in a few wells exposed to the inhibitory concentration of 2.5 µg/mL thiolutin. Single colonies from these wells were screened for their resistance against thiolution. Six mutant strains with significant resistance were selected from different mutant backgrounds and sent for whole-genome resequencing to SeqCentre (Pittsburgh, PA, USA). The reads were trimmed, mapped, and aligned with reference WT (NC_000964.3) genome sequence using CLC Genomics Workbench. Mutations that were present in the thiolutin-resistant mutants but not in the parent strain were determined (Table 1).

## SUPPLEMENTAL MATERIAL

Supplemental figures jb.00181-25-s0001.pdf Fig. S1, S2, and S3.

ASM does not own the copyrights to Supplemental Material that may be linked to, or accessed through, an article. The authors have granted ASM a non-exclusive, world-wide license to publish the Supplemental Material files. Please contact the corresponding author directly for reuse.
