# Supplementary material for: Reductive activation of the disulfide-containing antibiotic thiolutin is mediated by both bacillithiol and FAD-dependent disulfide reductases
Source: J Bacteriol. 2025 Jul 3;207(7):e00181-25. doi: 10.1128/jb.00181-25 (PMC12288471; doi:10.1128/jb.00181-25)
Supplement: Supplemental figures — Fig. S1, S2, and S3. [file jb.00181-25-s0001.pdf]

SUPPLEMENTARY INFORMATION

Reductive activation of the disulfide-containing antibiotic thiolutin is mediated by both bacillithiol and FAD-dependent disulfide reductases

Ahmed Gaballa, Yesha Patel, John D. Helmann†

Department of Microbiology, Cornell University, 370 Wing Hall, 123 Wing Drive, Ithaca, New York 14853-8101, USA

Fig. S1. Heatmap summarizing growth inhibition of *B. subtilis* WT and mutant strains in the presence of increasing concentrations of thiolutin.

Fig. S2. Heatmap summarizing growth inhibition of various *B. subtilis* parent strains and derived suppressor mutants.

Fig. S3. Growth of *B. subtilis* Δ*trxB amyE::P*$_{xyl}$*-trxB* in the presence of different concentrations of thiolutin.

A.

	0	0.156	0.25	0.3125	0.5	0.625	1	1.25	2	2.5
**CU1065**	100.0	90.2	88.8	78.7	53.7	29.7	1.6	1.6	1.2	1.5
bshC	100.0	94.7	86.7	83.9	76.1	64.3	26.0	3.1	1.2	1.1
bdr	100.0	102.1	94.7	94.0	63.8	38.5	1.6	0.8	0.9	2.4
brxAB	100.0	105.2	89.0	93.7	44.1	20.3	0.5	0.3	0.3	1.0
brxAB bdr	100.0	109.3	86.2	86.2	79.3	54.2	3.4	1.1	0.7	1.0
spx	100.0	93.2	92.2	89.9	81.1	66.8	21.7	14.3	3.7	2.9
spx P_{spac}-*spx*	100.0	89.4	85.0	79.3	57.3	27.3	2.5	1.1	1.3	1.5
spxP_{spac}-spx^{C10A}	100.0	76.8	79.8	74.2	75.8	71.0	29.8	9.0	2.3	2.1
spxP_{spac}-spx^{DD}	100.0	40.8	28.9	29.3	23.7	15.4	3.1	2.1	0.9	1.1
bshC spx	100.0	89.9	82.8	81.8	63.2	41.9	10.1	8.6	3.5	2.4
trxA P_{xyl}-*trxA*[1]	100.0	93.6	91.2	89.8	77.6	76.3	55.7	43.9	16.4	9.8
trxA P_{xyl}-*trxA*[2]	100.0	94.2	90.6	86.5	75.0	78.5	55.9	48.5	15.3	5.8
trxB P_{xyl}-*trxB*[1]	100.0	98.5	27.0	11.7	1.7	1.4	1.3	1.8	1.2	0.9
trxB P_{xyl}-*trxB*[2]	100.0	86.5	39.2	23.7	1.6	1.0	0.3	0.7	0.8	0.9
ahpC	100.0	104.6	91.4	93.6	64.1	34.3	0.8	0.6	1.0	1.2
ahpF	100.0	97.4	91.0	85.9	75.7	66.9	5.3	0.6	0.4	0.4
ahpF bshC	100.0	95.1	91.2	91.6	77.3	62.9	1.4	0.9	0.9	1.3
ahpF spx	100.0	95.8	94.4	94.3	89.8	84.5	48.6	28.0	5.1	2.8
ahpF bshC spx	100.0	90.4	81.7	82.1	55.1	51.5	24.3	9.0	2.6	1.8

B.

	0	0.156	0.25	0.3125	0.5	0.625	1	1.25	2	2.5
**CU1065**	100.0	97.2	109.1	107.0	106.3	90.7	7.8	1.6	1.4	1.4
bshC	100.0	91.4	99.6	98.5	104.6	108.3	62.4	21.5	1.4	1.3
bdr	100.0	109.6	121.6	117.3	122.5	112.9	3.3	1.0	1.1	3.7
brxAB	100.0	118.5	131.8	121.2	119.9	85.7	0.8	0.4	0.3	1.8
brxAB bdr	100.0	102.6	113.4	100.3	124.6	118.4	10.4	1.7	0.5	1.6
spx	100.0	95.8	96.4	98.8	105.8	114.7	98.1	70.0	4.3	3.3
spx P_{spac}-*spx*	100.0	100.3	102.8	102.3	94.1	84.3	18.2	2.7	0.8	1.4
spxP_{spac}-spx^{C10A}	100.0	94.8	96.2	79.5	92.7	80.3	50.8	45.1	1.5	1.4
spxP_{spac}-spx^{DD}	100.0	82.6	58.4	55.3	37.8	38.6	8.0	2.6	0.7	1.1
bshC spx	100.0	100.2	102.8	99.7	101.5	92.4	42.0	36.2	2.3	2.5
trxA P_{xyl}-*trxA*[1]	100.0	104.7	103.7	102.5	91.0	87.9	81.2	79.1	15.2	8.1
trxA P_{xyl}-*trxA*[2]	100.0	98.6	99.3	96.8	94.0	91.0	77.8	67.3	17.3	4.2
trxB P_{xyl}-*trxB*[1]	100.0	88.6	88.4	39.3	1.8	1.1	0.6	0.4	0.4	0.3
trxB P_{xyl}-*trxB*[2]	100.0	97.4	83.6	65.4	18.4	2.0	0.5	0.4	0.4	0.6
ahpC	100.0	104.8	103.7	100.6	108.2	87.5	0.9	0.8	1.1	1.8
ahpF	100.0	102.1	103.2	102.6	107.0	107.0	40.3	0.5	0.4	0.5
ahpF bshC	100.0	95.1	91.2	91.6	77.3	62.9	1.4	0.9	0.9	1.3
ahpF spx	100.0	95.8	94.4	94.3	89.8	84.5	48.6	28.0	5.1	2.8
ahpF bshC spx	100.0	90.4	81.7	82.1	55.1	51.5	24.3	9.0	2.6	1.8

C.

	0	0.156	0.25	0.3125	0.5	0.625	1	1.25	2	2.5
**CU1065**	100.0	90.9	102.2	97.9	101.3	101.8	29.0	8.9	1.7	1.9
bshC	100.0	89.0	91.7	92.5	97.0	100.8	67.9	31.8	1.4	1.5
bdr	100.0	97.9	103.0	102.0	108.4	107.3	4.1	1.0	1.0	3.6
brxAB	100.0	107.1	113.8	106.8	114.9	112.2	0.8	0.4	0.3	2.2
brxAB bdr	100.0	91.6	98.4	89.9	104.0	106.0	71.4	1.6	0.2	0.7
spx	100.0	93.4	92.3	91.4	96.5	107.7	113.7	87.7	18.9	3.2
spx P_{spac}-*spx*	100.0	95.4	95.2	95.4	95.7	94.8	34.1	31.5	0.9	1.8
spxP_{spac}-spx^{C10A}	100.0	93.8	93.5	91.0	92.6	91.7	68.1	65.3	1.4	1.6
spxP_{spac}-spx^{DD}	100.0	92.3	88.7	75.8	52.7	60.3	22.7	35.8	0.8	1.1
bshC spx	100.0	96.6	97.8	90.1	87.5	82.8	67.4	57.5	2.1	1.3
trxA P_{xyl}-*trxA*[1]	100.0	96.4	93.3	94.8	87.5	85.4	83.4	82.3	31.6	7.5
trxA P_{xyl}-*trxA*[2]	100.0	90.9	89.5	88.1	88.4	85.1	80.9	69.8	36.7	6.6
trxB P_{xyl}-*trxB*[1]	100.0	82.2	79.5	68.2	36.7	1.3	0.8	0.5	0.5	0.2
trxB P_{xyl}-*trxB*[2]	100.0	98.0	95.2	89.2	35.8	35.6	0.4	0.3	0.2	0.3
ahpC	100.0	98.3	97.8	94.4	101.5	98.0	0.9	0.8	1.2	1.8
ahpF	100.0	96.8	96.2	97.4	101.3	101.9	63.8	0.5	0.4	0.5
ahpF bshC	100.0	85.8	90.6	90.3	100.9	102.5	21.2	21.5	12.1	1.4
ahpF spx	100.0	83.7	83.0	80.9	84.1	85.3	93.0	89.5	25.2	2.5
ahpF bshC spx	100.0	85.0	81.5	78.8	78.0	82.6	80.3	75.3	2.0	1.1

Fig. S1. Heatmap summarizing growth inhibition of *B. subtilis* WT and mutant strains in the presence of increasing concentrations of thiolutin (left to right, in µg/mL as indicated beneath the heatmap). Results are the average of at least three biological replicates as shown in the main text growth curves for most strains. Values shown are cell density as a percent of the untreated control after (A) 12 hours, (B) 24 hours, and (C) 36 hours of growth at 37 ˚C. For the constructs with inducible *trxA* or *trxB*, [1] indicates 0.03% xylose and [2] indicates 0.3% xylose.

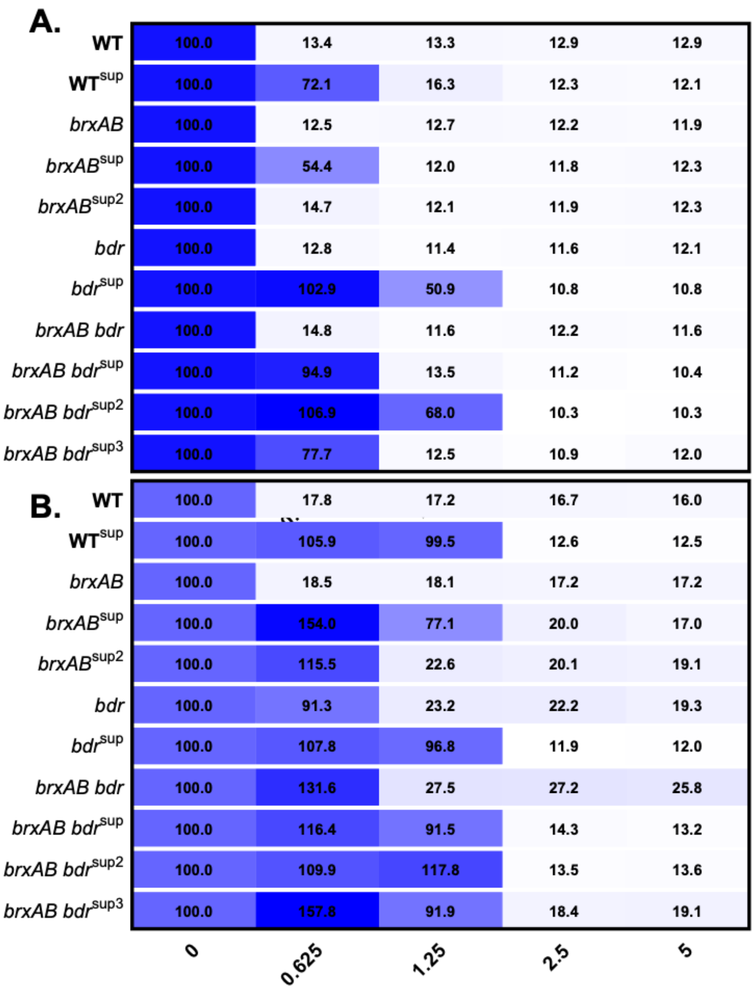

Fig. S2. Heatmap summarizing growth inhibition of various *B. subtilis* parent strains and derived suppressor mutants (as indicated by superscripts) in the presence of increasing concentrations of thiolutin (left to right, in µg/mL). Values shown are cell density as a percent of the control lacking thiolutin after (A) 12 hours and (B) 24 hours of growth in LB medium at 37 ˚C.

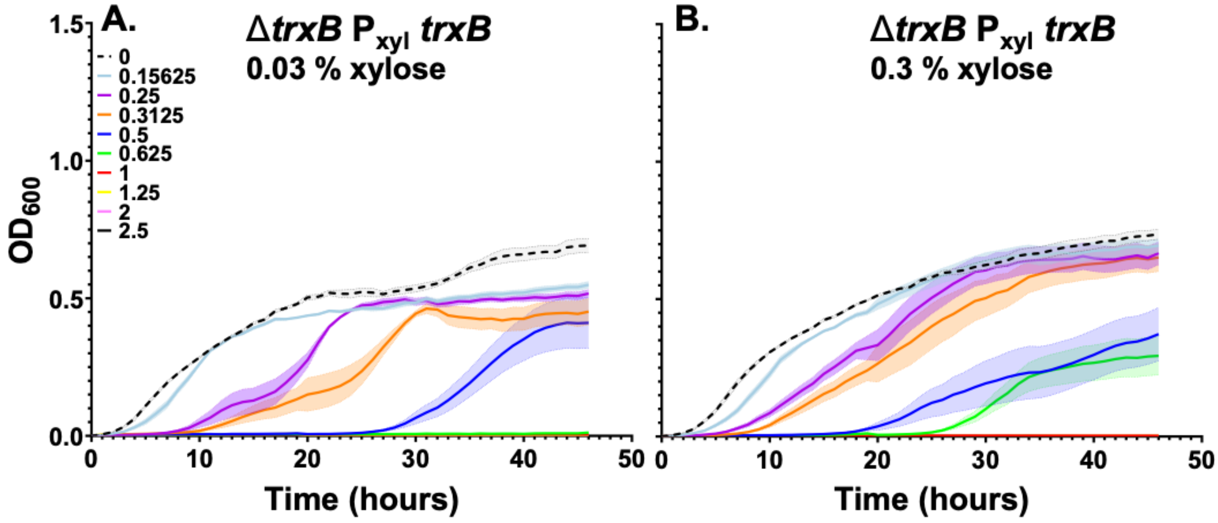

Fig. S3. Growth of *B. subtilis* Δ*trxB amyE::P$_{xyl}$-trxB* in the presence of (A) 0.03% xylose or (B) 0.3% xylose and different concentrations of thiolutin. Results are the average of at least three biological replicates, and the shaded region denotes the standard error of the mean.
